# Supplementary figures and images for: A Path-Based Analysis of Infected Cell Line and COVID-19 Patient Transcriptome Reveals Novel Potential Targets and Drugs Against SARS-CoV-2
Source: Front Immunol. 2022 Jul 1;13:918817. doi: 10.3389/fimmu.2022.918817 (PMC9284228; doi:10.3389/fimmu.2022.918817)

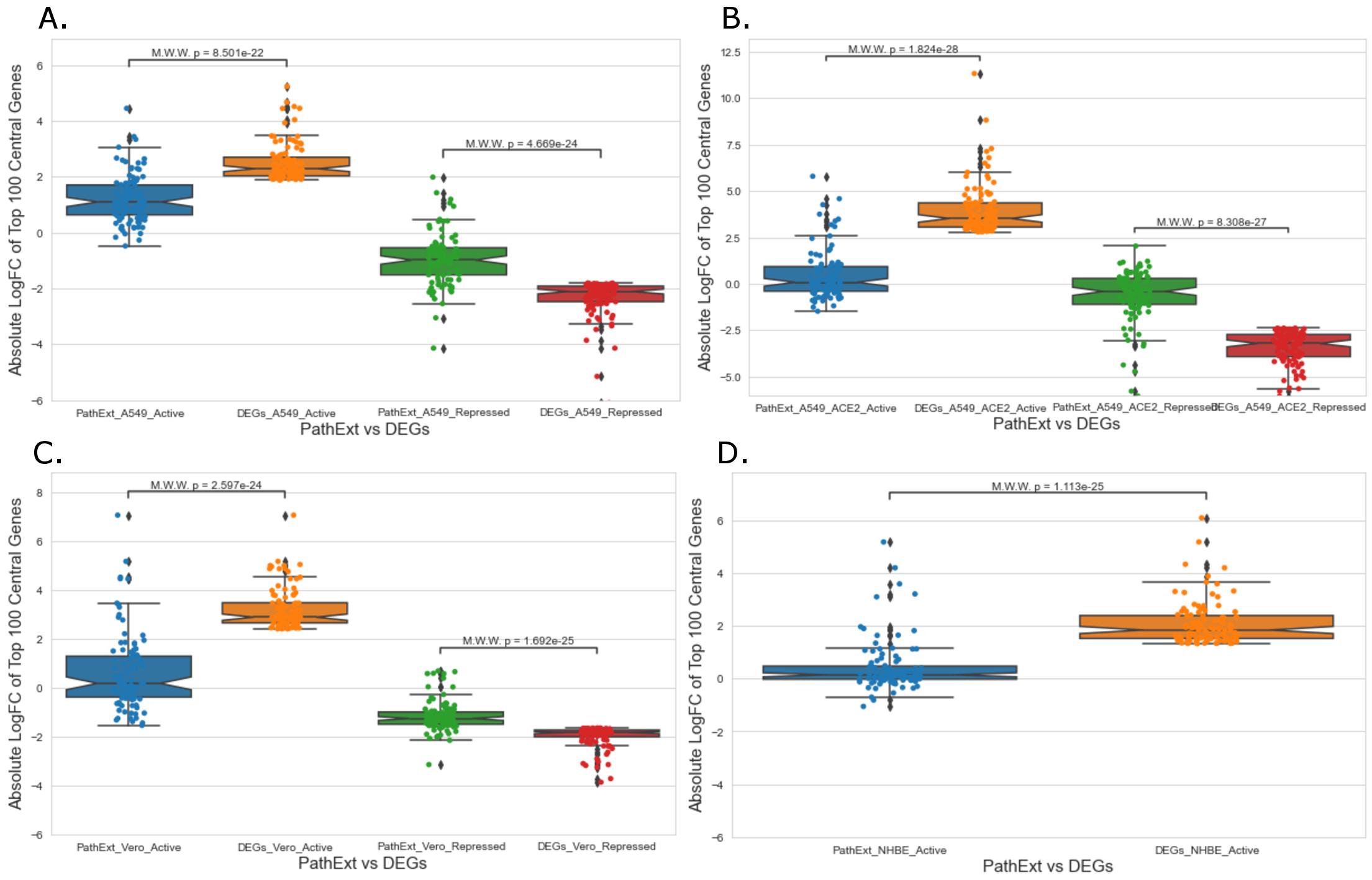

Supplement: Supplementary Figure 1 — Fold Change comparison of the top 100 frequent genes of PathExt TopNets and DEGs in various SC2 infected cell lines in (A) A549 cell line; (B) A549_ACE2 cell line; (iii) Vero Cell line; and (iv) NHBE cell line. [file Image_1.jpeg]

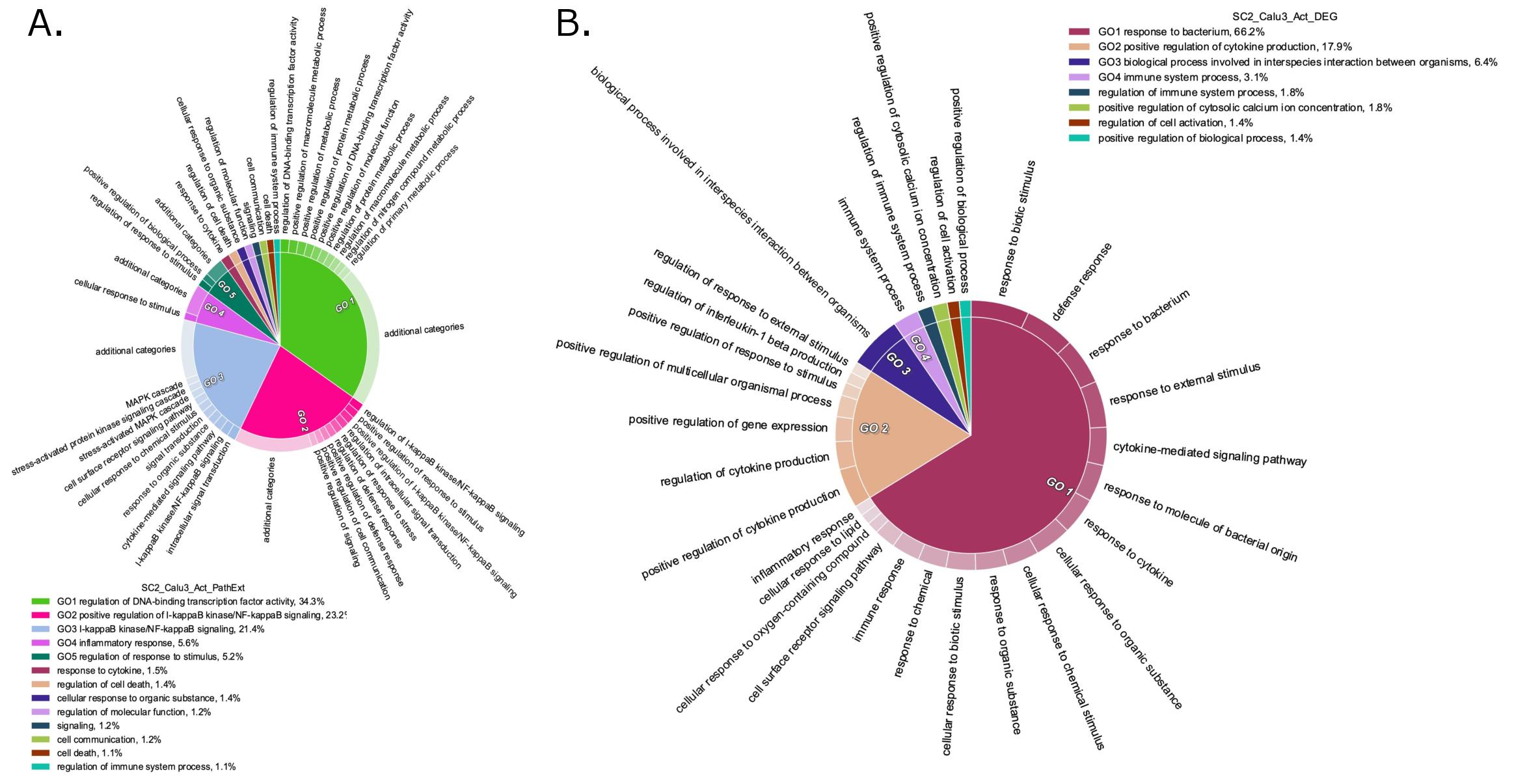

Supplement: Supplementary Figure 2 — Parent and child enriched pathways associated with upregulated DEGs in SC2 infected Calu3 cell line. Most frequent top 100 upregulated genes were obtained across patient PBMC data. Enriched biological processes was obtained by performing Gene Ontology study followed by parent child relationship, shown in the form of circular visualization plot. [file Image_2.jpeg]

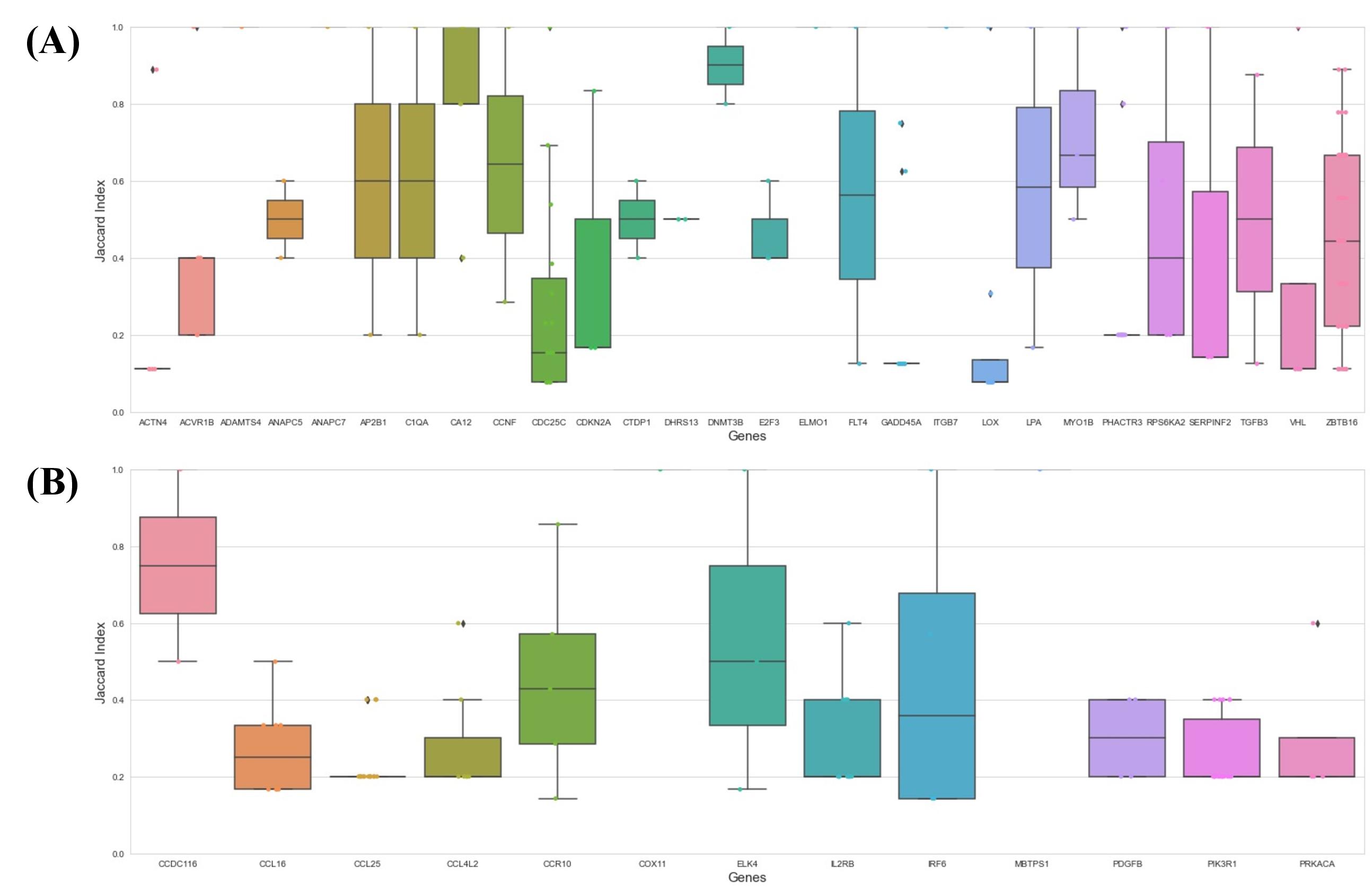

Supplement: Supplementary Figure 3 — Cross-sample overlap on TopNet neighbors of central genes. For each central genes (x-axis), the figures show the distribution of Jaccard Index of all sample-pair overlap between the TopNet neighbors of the central gene, for Activated TopNet for ICU patients (A) and non-ICU patients (B). [file Image_3.jpeg]

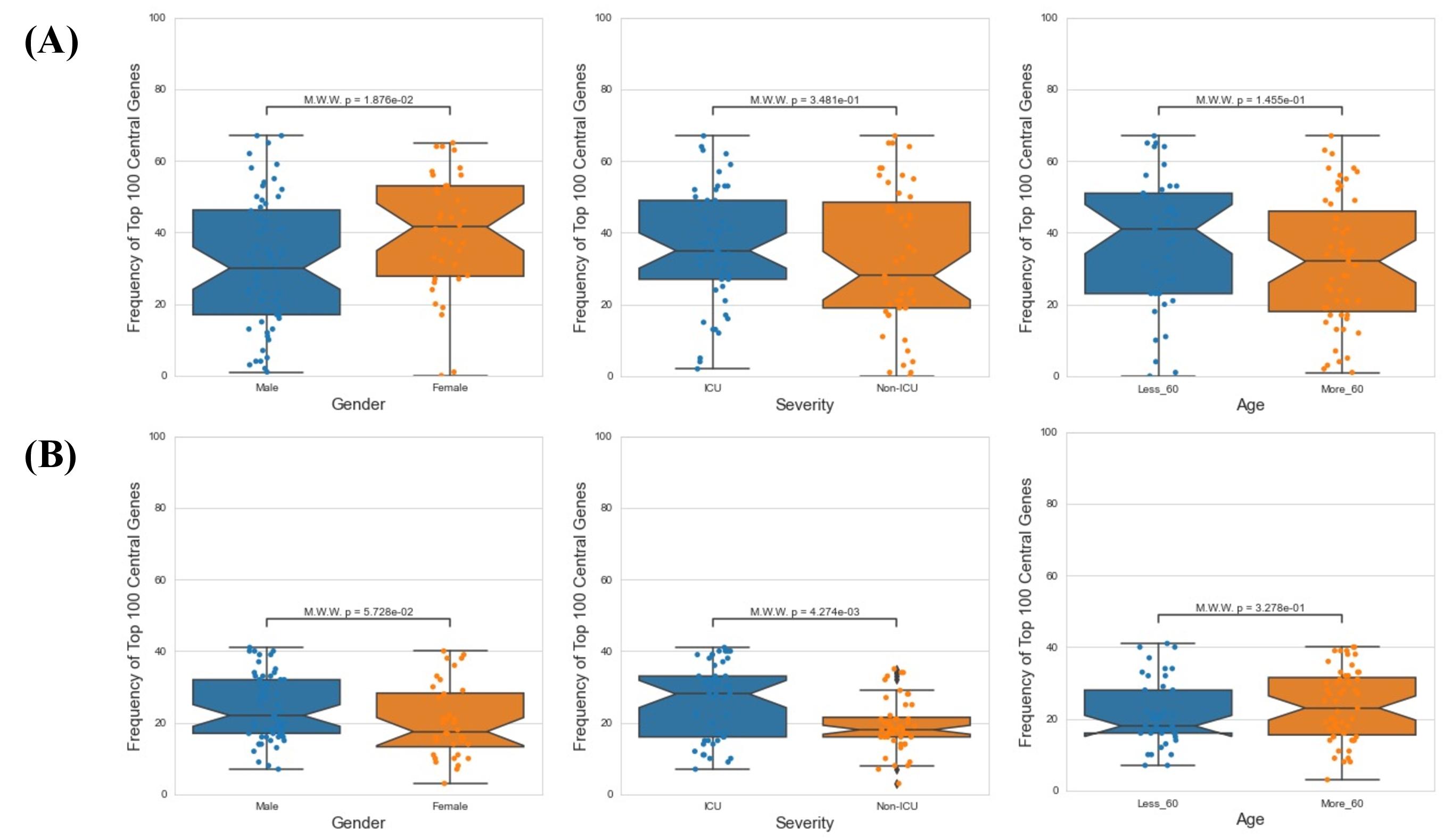

Supplement: Supplementary Figure 4 — Demographic features analysis. Mann Whitney Test was performed to check statistical significance between top 100 differentially expressed genes and various demographic features (age, sex and severity). In case of upregulated DEGs, “Gender” was found to be the only statistically group among activated TopNet genes (A) and in case of downregulated DEGs, “Severity” was found to be the only statistically group among repressed TopNet genes (B). [file Image_4.jpeg]

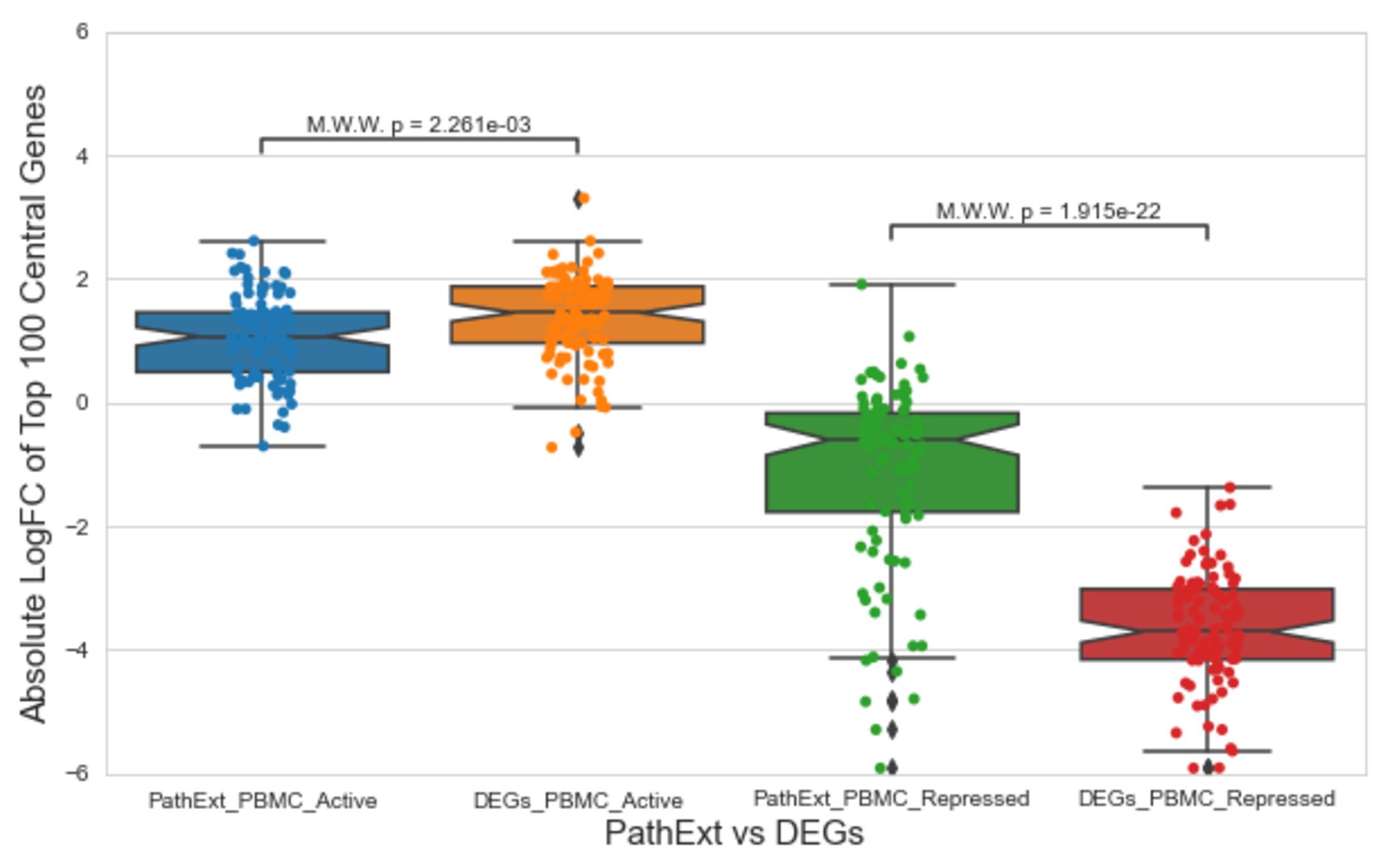

Supplement: Supplementary Figure 5 — Fold Change comparison of the top 100 frequent genes of PathExt TopNets and DEGs. LogFC comparison of the top 100 genes between PathExt and DEGs obtained from the SC2-infected patient PBMC data. Differential expression is estimated in infected relative to uninfected cells. Mean expression of the genes across 100 patients were considered while plotting [file Image_5.jpeg]

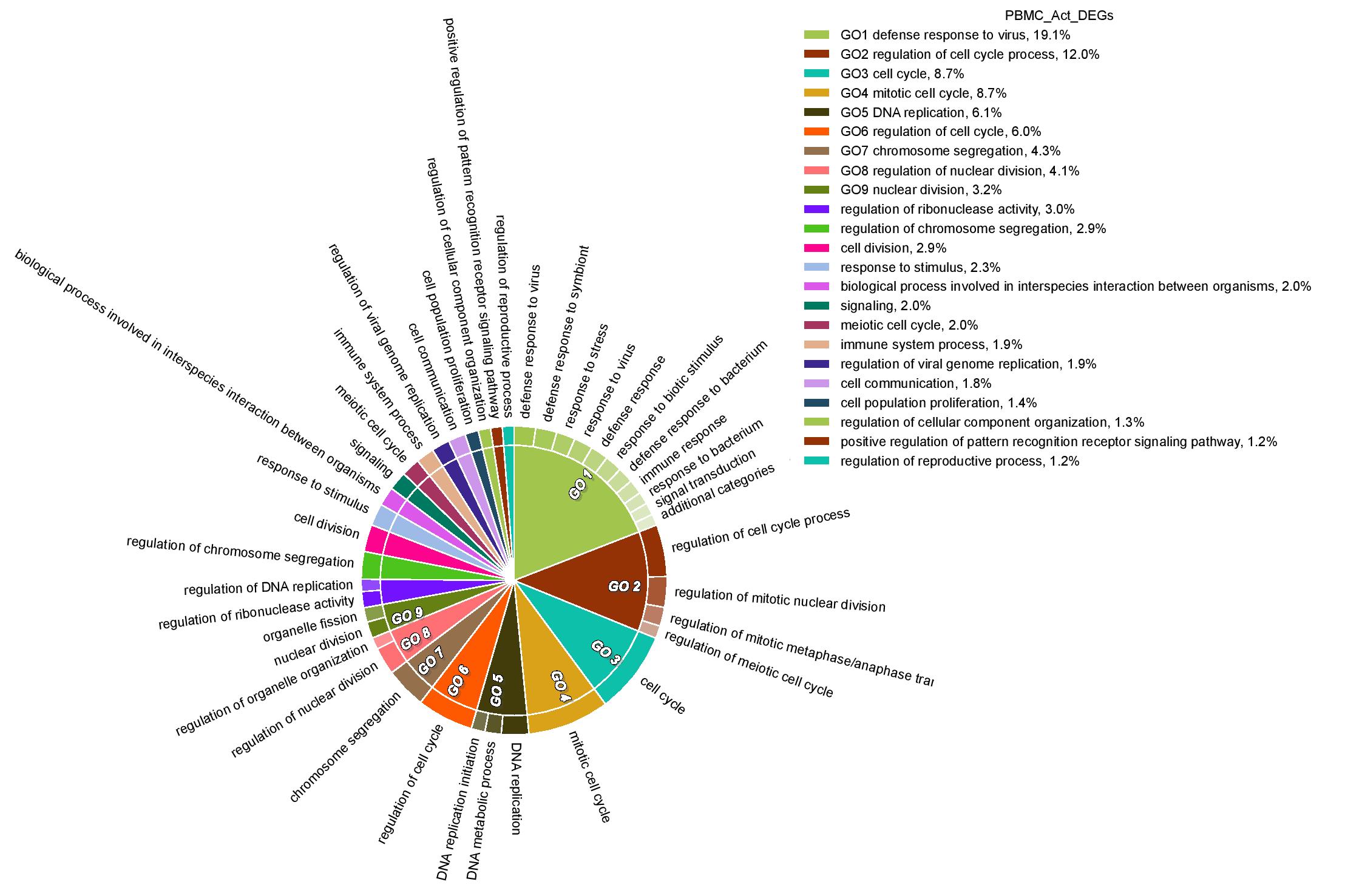

Supplement: Supplementary Figure 6 — Parent and child enriched pathways associated with upregulated DEGs in patient PBMC. Most frequent top 100 upregulated genes were obtained across patient PBMC data. Enriched biological processes was obtained by performing Gene Ontology study followed by parent child relationship, shown in the form of circular visualization plot. [file Image_6.jpeg]
